# The impact of health literacy on health-promoting lifestyle among community residents: the chain-mediating role of family health and physical activity

**DOI:** 10.3389/fpsyt.2024.1487274

**Published:** 2024-11-08

**Authors:** Yunxia Ma, Li Huang, Haodong Tian, Haowei Liu, Hanglin Yu, Hansen Li, Liya Guo

**Affiliations:** ^1^ College of Physical Education and Health, Yili Normal University, Xinjiang, China; ^2^ College of Physical Education, Southwest University, Chongqing, China; ^3^ School of Physical Education, Sichuan Agricultural University, Ya’an, China

**Keywords:** health literacy, healthy lifestyle, physical activity, family health, health-promotion lifestyle

## Abstract

**Background:**

Adopting health-promoting lifestyle (HPL) is crucial for improving overall well-being and reducing the risk of chronic diseases. The relationship between health literacy (HL) and HPL among Chinese community residents is complex, with potential mediating factors yet to be fully understood. Family health and physical activity (PA) may play significant roles in this relationship. This study aims to construct a chain mediation model to explore whether family health and PA mediate the effects of HL on HPL in Chinese community residents.

**Methods:**

Using the convenient sampling method, 1,072 Chinese community residents were selected for a cross-sectional study. All participants completed a self-report questionnaire that collected demographic information, as well as data from the Health Literacy Scale Short-Form (HLS-SF12), Family Health Scale Short-Form (FHS-SF), Physical Activity Rating Scale (PARS-3), and Health Promoting Lifestyle Profile-II Revise (HPLP-II R). Data were analyzed using SPSS 26.0, with mediation analysis performed using the SPSS PROCESS macro.

**Results:**

There were significant pairwise correlations between HL, family health, PA, and HPL (*p* < 0.01). HL was directly linked to HPL (effect = 0.442; SE = 0.025; 95% CI: 0.392, 0.491). Additionally, three indirect pathways were identified: family health independently mediated 6.02% of the effect (effect = 0.032; SE = 0.010; 95% CI: 0.013, 0.051), PA also independently mediated 9.02% of the effect (effect = 0.048; SE = 0.010; 95% CI: 0.030, 0.068), and a combined chain mediation through both family health and PA accounted for 1.88% of the effect (effect = 0.010; SE = 0.003; 95% CI: 0.005, 0.017).

**Conclusion:**

HL not only has a direct impact on promoting HPL but also influences it indirectly through the mediating roles of family health and PA. These insights elucidate the mechanisms by which HL affects HPL, providing valuable theoretical guidance for the development and implementation of effective strategies to encourage healthy lifestyle practices.

## 1 Introduction

Lifestyle is recognized as the most significant factor affecting the health of people (1). According to the *Report on Nutrition and Chronic Diseases of Chinese Residents (2020)*, unhealthy lifestyles are still widespread among Chinese residents, which closely linked to the increasing prevalence of overweight and obesity and a continued rise in rates of major chronic diseases. Health-promoting lifestyle (HPL) refers to actions that individuals take the initiative to pursue that could benefit their health (2), and its six components include health protection and health promotion activity, proper nutrition, health responsibility, stress management, interpersonal relationships, and spiritual growth (3). Currently, HPL has been highly emphasized, as it exhibited effectiveness in lowering the incidence and mortality rates of various chronic diseases like cardiovascular diseases, cancer, and chronic respiratory diseases, and enhancing life expectancy among Chinese adults (4). And these merits highlighted HPL’s crucial role in preventing diseases, reducing pathogenicity, improving the quality of life, and decreasing the burden of health care in societies (5).

Health literacy (HL) is defined as the cognitive and social skills which determine the motivation and ability of individuals to gain access to, understand, and use information in ways that promote and maintain good health (6). However, although the positive correlation between HL and HPL has been established (7, 8), the underlying mechanisms governing their interplay remain unclear (9), preventing causal inferences. Understanding how HL impacts HPL is crucial for explaining the formation of HPL (10), thereby informing the development of more effective health promotion strategies.

The relationship between HL and HPL may be mediated by various factors, with family health being particularly significant. Family health is defined as a resource at the level of the family unit that develops from the intersection of the health of each family member, their interactions and capacities, as well as the family’s physical, social, emotional, economic, and medical resources (11). It emphasizes the interactions and communication among family members who share the same ecological niche, which are dynamically shaped by family function, environment, and structure (12). Member’s HL is considered an internal resource within the family, and has been shown to be positively correlated with family function (13). Improving HL not only helps to form accurate health concepts and attitudes but also enhances the ability to master health skills and increase access to healthcare information. Moreover, higher HL can lead to reduced family medical expenses and overall improvement in family health (14). In addition to the relationship with HL, family health is also of great importance in fostering HPL (15). There are a range of mechanisms underlying relationships between family and HPL, including promoting health-seeking or health treatment behaviors providing access, opportunities, and resources for a range of health behaviors (16). Conclusively, family health may significantly mediate the influences of HL on the formation on HPL, as higher HL enhances family function and facilitates access to necessary resources, ultimately leading to more effective HPL and improved overall well-being. Besides family health, physical activity (PA) may serve as another key factor linking HL to HPL. There is a strong relationship between HL and PA, with higher levels of HL being a strong predictor of more frequent PA (17). This suggests that enhancing residents’ HL can significantly increase their levels of PA. Furthermore, there was a positive correlation between HPL and levels of PA (18). Mašina et al. found that low-intensity PA was correlated with the HPL subscales of health responsibility and spiritual growth (19). The identical experiential foundation of PA and HPL may help explain their correlation and mutual influence (20). Specifically, individuals can develop a sense of health responsibility, emotional growth, positive interpersonal relationships, nutritional management, and stress management skills through PA. At the same time, these experiences can also originate from HPL, indicating a natural connection between PA and HPL.

Additionally, there is an obvious impact of family health found on individual’s PA. The family health serves as the most direct external stimulus for motivating exercise awareness among its members, with various family-related factors influencing an individual’s PA. Existing research has already focused on some specific factors of family dynamics, for example, Morrissey et al. found that family support, including encouragement and active participation from family members, significantly enhances adolescents’ PA levels (21). Moreover, the complexity of family structure also directly influences the exercise behaviors of its members. A stable family structure, coupled with harmonious relationships, can promote greater participation in physical activity (22). Similarly, Zhang et al. found that family environment plays a significant role in shaping the PA habits of adolescents (23). However, the studies that examining the overall impact of family health on PA are still limited. In fact, the connotation of family health is richer and deeper than that of family function, and it is more applicable to the field of health (24). Therefore, it is necessary to comprehensively reveal the role of both family health and PA in the interaction between HL and HPL.

**Figure 1:**
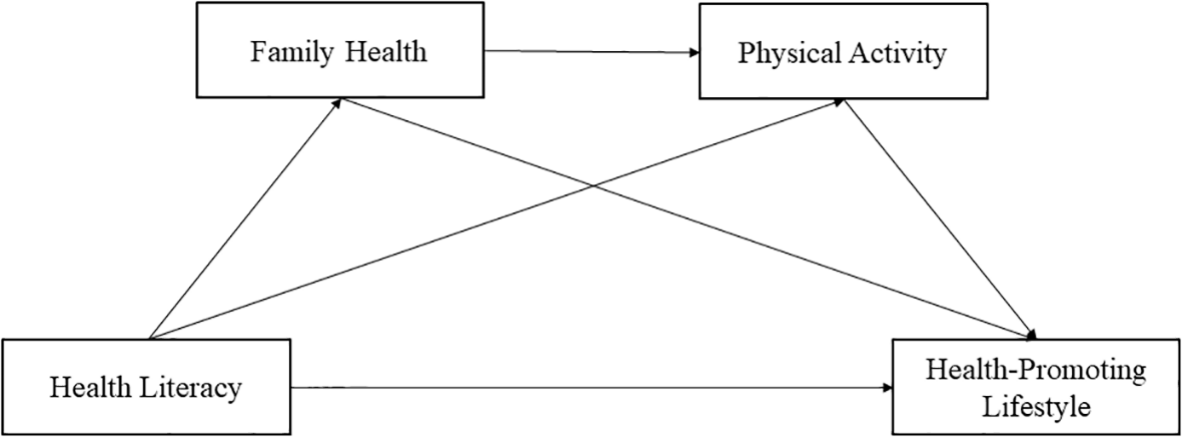
Hypothetical model diagram.

Given existing evidence, our study aims to explore the pathways through which HL can promote a HPL, providing guidance for interventions aimed at enhancing HPL. we hypothesized that the association between HL and HPL is mediated by family health and PA. Our model is shown in **Figure 1**.

## 2 Methods and participants

### 2.1 Procedure and participants

An online cross-sectional study was conducted from August 2023 to November 2023 using the convenience sampling method. This study utilized the popular survey software in China (https://www.wjx.cn/). A total of 1,238 questionnaires were collected, of which 166 invalid questionnaires were excluded based on the following criteria: (1) respondents younger than 18 years old; (2) incomplete responses, defined as questionnaires with more than three unanswered items; (3) patterned responses, identified by repeated selection of the same option across multiple items; (4) obvious inconsistencies in responses, detected through validation questions designed to identify non-serious or deceptive responses; (5) completion time less than 270 seconds, which was deemed insufficient to complete the survey with thoughtful consideration; and (6) non-community residents, such as university students who primarily reside on campus and not within the community. Ultimately, 1,072 valid questionnaires were included, yielding an effective response rate of 86.59%.

To ensure data integrity, we monitored participants’ devices and IP addresses, allowing each user to complete the survey only once. The system also tracked the completion times of all submissions, which helped to identify and exclude suspicious or duplicate responses. All collected data were treated with strict confidentiality measures, and no personal identifiers such as names were collected. Participants were required to sign an informed consent form before proceeding with the survey, and all data were stored in an encrypted database to maintain confidentiality.

The sample covered the four major economic regions of China, primarily distributed in the Western region (e.g., Chongqing, Sichuan, Inner Mongolia), the Eastern region (e.g., Beijing, Guangdong, Shandong), and the Central region (e.g., Henan, Hunan, Shanxi). Among the respondents, 685 were male (63.9%) and 387 were female (36.1%). Due to the use of online convenience sampling, there were age distribution differences, with the sample mainly consisting of young adults (18-44 years) at 565 (52.7%), middle-aged adults (45-59 years) at 418 (39%), and older adults (60 years and above) at 89 (8.3%).

This study was approved by the Ethics Committee of Southwest University Hospital (SWU-ETF-2023-07-17-011).

### 2.2 Measurements

#### 2.2.1 Health literacy

Health literacy was measured using the Chinese version of Health Literacy Scale Short-Form (HLS-SF12), which was adapted by Duong et al. (25), and translated and introduced into China by Sun et al. (26). The HLS-SF12 consists of 12 items across 3 dimensions: health care (4 items), disease prevention (4 items), and health promotion (4 items). The scale employs a 4-point Likert scoring system with response options ranging from very difficult to very easy (1 = very difficult, 2 = difficult, 3 = easy, 4 = very easy). The health literacy index is calculated as follows: Health Literacy Index = (Mean - 1) * (50/3), with index scores ranging from 0 to 50, where higher scores indicate higher health literacy. In this study, the Cronbach’s α was 0.933.

#### 2.2.2 Health-promoting lifestyle

Health-promoting lifestyle was measured using the Chinese version of the Health Promoting Lifestyle Profile-II Revise (HPLP-II R), which was cross-culturally adapted by Cao et al. (27) from the Taiwanese version of the Health Promoting Lifestyle Profile(HPLP-II) (28). The scale consists of 40 items, which was categorized into six dimensions: interpersonal relations (5 items), nutrition (6 items), health responsibility (11 items), physical activity (8 items), stress management (5 items), and spiritual growth (5 items). Responses to each item are scored on a scale from never (1 point) to always (4 points). The total score ranges from 40 to 160, with higher scores indicating a higher level of health-promoting lifestyle. In this study, the Cronbach’s α was 0.956.

#### 2.2.3 Family health

Family health was measured using the Family Health Scale Short-Form (FHS-SF), which developed by Crandall et al. (29) and translated and culturally adapted into Chinese by Wang et al. (30). This scale serves as a tool to evaluate family health in China and is suitable for adults aged 18 and above. The FHS-SF measures family health function through 10 items across 4 dimensions: family/social/emotional health processes (items 1, 2, 5), family healthy lifestyle (items 3, 4), family health resources (items 6, 9, 10), and family external social support (items 7, 8). The FHS-SF uses a 5-point Likert scale ranging from strongly disagree to strongly agree. Items 6, 9, and 10 are reverse scored, while the remaining items are positively scored. The total score of all items is calculated, with higher scores indicating better family health status. In this study, the Cronbach’s α was 0.735.

#### 2.2.4 Physical activity

Physical activity level was measured using the Physical Activity Rating Scale (PARS-3) (31), which includes 3 items: intensity, duration, and frequency. Each item is evaluated from 1 to 5 points, and the total score of PA is computed by the equation below: intensity × (duration −1) × frequency, of which the score ranges from 0 to 100. The PA level was divided into low, moderate and high categories: low ≤ 19 points, 20 ≤ moderate ≤ 42 points, and high ≥43 points (32). This scale has been frequently used in Chinese populations (33, 34). In this study, the Cronbach’s α was 0.696.

### 2.3 Statistical analyses

The statistical analysis was conducted using IBM SPSS 26.0 (35) and the PROCESS plug-in (36) developed by Hayes. Initially, we tested common method bias using common latent factor. Then, correlation analysis and regression analysis were sequentially conducted to explore the relationship between HL, HPL, family health and PA. Furthermore, the mediation effects between HL and HPL were tested using the bias-corrected percentile Bootstrap method with 5000 times of repeated sampling (37). The statistical significance of mediation effects was evaluated based on the 95% confidence interval generated by the bootstrapping (38, 39). In this study, sex and age was set as a control variable due its known effects on the variables of interest (40).

## 3 Results

### 3.1 Common method bias

The Harman one-way test was conducted to assess common method bias. Results from the unrotated exploratory factor analysis showed that the first factor accounted for 30.76% of the variance, which is below the critical threshold of 40%. This suggests that there is no significant common method bias in this study.

### 3.2 Descriptive analysis and correlations between overall variables

The averages and standard deviations of the participants’ HL, HPLP, family health and PA were computed. The correlations between the variables of interest are shown in **Table 1**. HL was significantly positively correlated with HPL (r = 0.554, *p* < 0.01), family health (r = 0.366, *p* < 0.01), and PA (r = 0.211, *p* < 0.01). HPL was significantly positively correlated with family health (r = 0.306, *p* < 0.01) and PA (r = 0.366, *p* < 0.01). Family health was also positively correlated with PA (r = 0.164, *p* < 0.01).

**Table 1:** Descriptive statistics and correlation analysis between core variables.

Variables	M ± SD	HL	HPL	Family Health	PA
HL	35.93 ± 7.97	1			
HPL	110.04 ± 18.21	0.554**	1		
Family Health	38.41 ± 6.46	0.366**	0.306**	1	
PA	26.56 ± 21.50	0.211**	0.366**	0.164**	1

SD denotes standard deviation, ** *p* < 0.01.

### 3.3 Regression analyses

**Table 2** shows the regression analyses controlled for sex and age. HL significantly predicted HPL (β =0.532, *p* < 0.001). After incorporating family health and PA into the model, HL still significantly predicted HPL (β=0.442, *p* < 0.001). HL also significantly predicted family health (β=0.368, *p* < 0.001) and PA (β=0.177, *p* < 0.001). Family health significantly predicted both HPL (β=0.086, *p* < 0.001) and PA (β=0.105, *p* < 0.001). Finally, PA significantly predicted HPL (β=0.271, *p* < 0.001).

**Table 2:** Regression analysis of the relationship between health literacy and health-promoting lifestyle.

Regression Equation	Predictor Variable	Fitting index				Significance	
Outcome Variable		R	R^2^	F	β	SE	t
HPL	Sex				0.035	0.051	0.676
	Age				0.012	0.038	0.317
	HL	0.555	0.308	158.08***	0.532	0.025	21.727***
Family Health	Sex				0.070	0.060	1.175
	Age				0.234	0.045	5.248***
	HL	0.396	0.157	66.369***	0.368	0.028	12.962***
PA	Sex				-0.532	0.059	-9.039***
	Age				0.035	0.045	0.784
	HL	0.348	0.121	36.628***	0.177	0.030	5.848***
	Family Health	–	–	–	0.105	0.030	3.460***
HPL	Sex				0.171	0.050	3.402***
	Age				-0.024	0.037	-0.658
	HL	0.622	0.386	134.27***	0.442	0.025	17.529***
	Family Health	–	–	–	0.086	0.025	3.458***
	PA	–	–	–	0.271	0.025	10.761***

*** *p*<0.001.

### 3.4 Mediation effect testing

**Figure 2:**
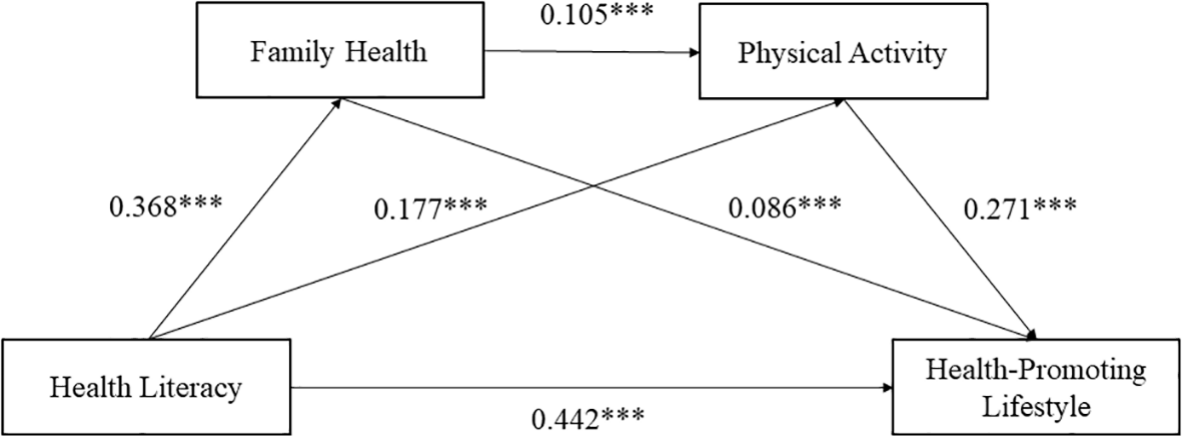
The chain mediating effect of health literacy and health-promoting lifestyle. ***p ≤ 0.001.

The mediation effect test results (**Table 3**) revealed that both family health and PA mediated variables of the influence of the HL and HPL, with a total mediation effect value of 0.090, 95% CI [0.066, 0.117]. Our study found that the mediating effect of family health and PA can be achieved through the following three paths. Path1: HL → family health → HPL, with effect value of 0.032, 95% CI [0.013 0.051], excluding 0, indicating that the mediating effect of the pathway was significant. Path 2: HL → PA→ HPL, with effect value of 0.048, 95% CI [0.030, 0.068], excluding 0, indicating that the mediating effect of the pathway was significant. Path 3: HL → family health → PA → HPL, with effect value of 0.010, 95% CI [0.005, 0.017], excluding 0, indicating that the mediating effect of the pathway was significant. Although the effect value is small, this pathway remains statistically significant. Overall, the hypotheses were partially supported (**Figure 2**).

**Table 3:** Mediating effects of family health and physical activity.

Pathways	Effect	Bootstrap SE	Bootstrap CI		Efficiency Ratio
			LLCI	ULCI	
HL→ HPL	0.442	0.025	0.392	0. 491	83.08%
HL→ Family health→ HPL	0.032	0.010	0.013	0.051	6.02%
HL→ PA→ HPL	0.048	0.010	0.030	0.068	9.02%
HL→ Family health→ PA→ HPL	0.010	0.003	0.005	0.017	1.88%
Total indirect effect	0. 090	0.013	0.066	0.117	16.92%
Total indirect effect	0.532	0.025	0.484	0.580	

## 4 Discussion

This study proposed a conceptual model to reveal the effect of HL on the HPL and the mediating role of family health and PA among Chinese residents. The results indicate that HL significantly impacts HPL positively and this effect is partly mediated indirectly through family health and PA. In recent years, improving the HL of Chinese community residents and encouraging healthy lifestyle have become key objectives in the “Healthy China initiative” (41), as well as the core elements in enhancing public health. Our analysis revealed a significant positive correlation between HL and HPL. Furthermore, our results support the previous findings that HL is a significant positive predictor of HPL (42–44). This implies that among Chinese residents, individuals with higher level of HL are more likely to adopt HPL. In health belief model, perceived susceptibility to and severity of illness or its sequelae, perceived benefits of taking a particular action minus perceived costs or barriers to action, and health motivation are emphasized (45). This may help explaining our findings because higher levels of HL signify a better understanding of health knowledge and an improved ability to choose accurate information, thereby facilitating HLP.

To further explore the pathways between HL and HPL, we introduce family health in our model. Existing evidence has consistently demonstrated the important role of family health in promoting HPL (46). However, to our knowledge, there are limited studies revealing the impact of HL on family health, this study is the first to reveal it as an independent mediator between HL and HPL. In addition, previous studies mainly examined specific dimensions (e.g. family healthy lifestyle (47)) of family health on HPL, while we treated its total score as one variable to explore its overall impact. The connection between family and HPL is mediated through various mechanisms, such as encouraging health-seeking and treatment behaviors, as well as offering access, opportunities, and resources for engaging in diverse health-related behaviors (16). Family health, with its broader scope of social support, internal interactions, and resources, promotes healthier behaviors more effectively than family function or family climate alone (48). Furthermore, the HL of individual family members significantly impacts the overall health of the family unit. As HL improves, it enhances family health, thereby increasing the self-efficacy of family members and facilitating the development and maintenance of healthy behaviors (49). Thus, the level of family health plays a vital role in shaping individual healthy lifestyles, demonstrating significant associations with both HL and HPL. Meanwhile, PA also serves as an independent mediator in the relationship between HL and HPL. HL might play a role in motivating people to become or stay physically active, as higher HL leads to greater awareness of the Active Guide and a higher proportion of individuals engaging in elevated physical activity levels (50). As a primary determinant of health behavior, PA can initiate a chain reaction that fosters HPL. PA initiates a cascade of HPL, including health responsibility, fostering spiritual growth, cultivating harmonious interpersonal relationships, maintaining a balanced diet, and managing stress effectively (51, 52). Kendzierski et al. (53) revealed the chain reaction mechanism between PA and HPL through the concept of exercise schemata. The purpose of exercise schemata is to shape physical appearance, maintain vitality, engage in PA, and promote health. This concept underpins the fundamental logic linking PA to HPL. However, it is important to note that as the amount of exercise increases, its benefits also increase, but when the amount of exercise exceeds a certain level, it may have a negative impact on an individual’s physical an d mental health. In fact, the relationship between PA and various indicators of physical and mental health may follow an inverted U-curve rather than a linear trend (54). Therefore, it is crucial to tailor physical activity plans to different exercise groups and types, rather than simply increasing duration, frequency, and intensity. Therefore, it is essential to design personalized physical activity plans tailored to individual characteristics, such as gender and age, to optimize the effectiveness of the activity.

Additionally, our study found that HL not only has a direct positive impact on HPL but also influences it indirectly through the mediating roles of family health and PA. According to the spillover hypothesis theory, the emotional attitudes, cognitive processes, and behavior patterns of family members influence one another through interactions. For example, parental support, involvement, communication, limit setting, and autonomy will influence children’s behaviors (55). When parents actively engage in PA, they can inspire their children to replicate and adopt similar behaviors, thereby establishing corresponding habits (56). Furthermore, John et al. found that family support can promote and maintain PA (57), while Lam et al. (58) found that family members’ levels of PA correlates with family lifestyle. Based on this evidence, it can be concluded that family-related factors, such as atmosphere and support, positively influence individuals’ engagement in physical exercise. These elements, which are integral to family health, elucidate the internal mechanisms through which family health impacts PA. Notably, although the chain mediating effect size was relatively small in this study, both family health and PA demonstrated independent effects on individuals’ HPL. This highlights the potential of family support and PA to enhance individuals’ HPL, providing empirical evidence for their roles as mediators in fostering a healthy lifestyle. However, our study also indicates that other factors, such as specific dimensions of family health, individual mental health, and social support, may also influence the development of HPL. Future research should explore these additional factors to gain a more comprehensive understanding of the mechanisms underlying HPL.

## 5 Limitations

Due to the convenience of online sampling, our study includes a limited number of elderly participants. Future research should incorporate offline surveys to increase the sample size of elderly participants. Additionally, the samples are predominantly drawn from the eastern and western economic regions, with relatively few from the Northeast, which may introduce regional spatial bias into the findings. To mitigate this bias, future studies should aim for more balanced sample collection across different regions and age groups.

Furthermore, this study does not control for key socio-economic and psychological factors such as education level, income, occupation, and mental health status, which are known to significantly influence health literacy and health-promoting lifestyle choices. Their exclusion could affect the study’s reliability and generalizability, particularly regarding the relative impact of health literacy. The primary reason for not including these factors was the limitation of the available data in our dataset, as we were unable to collect detailed socio-economic and psychological information. This restricted our ability to control for these variables. Future studies should incorporate these factors to provide a more comprehensive understanding of the relationship between health literacy and health-promoting lifestyles. Additionally, conducting sensitivity analyses to estimate how these confounding variables might alter the findings would further strengthen the robustness of the conclusions.

Family health encompasses four dimensions, and PA can be categorized into three levels. However, due to the limitations of the statistical methods used, our study does not specify which dimensions of family health or levels of PA most strongly influence the HPL. Future research should explore the effects or dose-response relationships of these different dimensions and levels, potentially using various measurement tools, to provide a more precise and effective reference for HPL.

Lastly, our study employs a cross-sectional survey design. To gain deeper insights, future research could consider longitudinal data collection across multiple regions and time points, or implement intervention measures to further explore the impacts of HL, family health, and PA on HPL.

## 6 Conclusion

HL, family health, PA, and HPL were all significantly positively correlated. HL not only directly influences HPL but also exerts an indirect effect through the mediating roles of family health and PA. Specifically, three mediating pathways were identified: one through family health, another through PA, and a combined pathway involving both family health and PA. Although the chain mediating effect was relatively small, its significance highlights the independent contributions of family health and PA in promoting individual HPL. Future research should investigate additional mediating factors and influences to deepen our understanding of the mechanisms that drive the formation of HPL.

## Data Availability

The raw data supporting the conclusions of this article will be made available by the corresponding author, without undue reservation.
